# Joint Time-Reversal Space-Time Block Coding and Adaptive Equalization for Filtered Multitone Underwater Acoustic Communications

**DOI:** 10.3390/s20020379

**Published:** 2020-01-09

**Authors:** Lin Sun, Ming Yan, Haisen Li, Yanjie Xu

**Affiliations:** 1 School of Physics and Physical Engineering, Qufu Normal University, Qufu 273165, China; 2 College of Underwater Acoustic Engineering, Harbin Engineering University, Harbin 150001, China; xuyanjie0127@163.com; 3 School of Mechanics and Engineering Science, Shanghai University, Shanghai 200444, China; SYMing508@shu.edu.cn

**Keywords:** underwater acoustic communications, filtered multitone, time-reversal space-time block coding, adaptive equalization

## Abstract

Underwater acoustic (UWA) sensor networks demand high-rate communications with high reliability between sensor nodes for massive data transmission. Filtered multitone (FMT) is an attractive multicarrier technique used in high-rate UWA communications, and can obviously shorten the span of intersymbol interference (ISI) with high spectral efficiency and low frequency offset sensitivity by dividing the communication band into several separated wide sub-bands without guard bands. The joint receive diversity and adaptive equalization scheme is often used as a general ISI suppression technique in FMT-UWA communications, but large receive array for high diversity gain has an adverse effect on the miniaturization of UWA sensor nodes. A time-reversal space-time block coding (TR-STBC) technique specially designed for frequency-selective fading channels can replace receive diversity with transmit diversity for high diversity gain, and therefore is helpful for ISI suppression with simple receive configuration. Moreover, the spatio-temporal matched filtering (MF) in TR-STBC decoding can mitigate ISI obviously, and therefore is of benefit to lessen the complexion of adaptive equalization for post-processing. In this paper, joint TR-STBC and adaptive equalization FMT-UWA communication method is proposed based on the merit of TR-STBC. The proposed method is analyzed in theory, and its performance is assessed using simulation analysis and real experimental data collected from an indoor pool communication trial. The validity of the proposed method is proved through comparing the proposed method with the joint single-input–single-output (SISO) and adaptive equalization method and the joint single-input–multiple-output (SIMO) and adaptive equalization method. The results show that the proposed method can achieve better communication performance than the joint SISO and adaptive equalization method, and can achieve similar performance with more simpler receive configuration as the joint SIMO and adaptive equalization method.

## 1. Introduction

Activities in underwater environments such as environmental monitoring, equipment location and assisted navigation have promoted the development of underwater acoustic (UWA) sensor networks, where high-rate communications with high reliability are required for massive data transmission between sensor nodes [1,2,3]. The UWA channel is a typical frequency-selective fading channel [4,5], and therefore when single-carrier (SC) modulation is used, a highly complex intersymbol interference (ISI) suppression technique must be adopted at the receiver to deal with massive ISI caused by severe multipath propagation [6,7,8,9]. To reduce the complexity of ISI suppression, multicarrier (MC) modulation has been used in UWA communications [9,10,11,12,13,14,15,16,17,18,19,20]. Orthogonal frequency division multiplexing (OFDM) as a MC technique is the most widely used in UWA communications [12,13,14,15]. In OFDM, the effect of multipath is restricted within one symbol interval by dividing the communication band into many sub-bands with bandwidth narrower than coherent bandwidth, and therefore the ISI can be avoided. However, OFDM is sensitive to frequency offset due to the strongly overlapping sub-bands with narrow bandwidth, and therefore the complex intercarrier interference (ICI) suppression technique must be exploited in OFDM-UWA communications. Filtered multitone (FMT) modulation is a multicarrier technique that splits the communication band into several separated wide sub-bands without guard bands [16,17,18,19,20]. Compared with SC, FMT can obviously reduce the complexity of ISI suppression by band splitting. Compared with OFDM, FMT is not sensitive to frequency offset due to non-overlapping sub-bands with much wider bandwidth. Therefore, as the compromise between MC and OFDM, FMT has been applied to UWA communications in the last 10 years.

In FMT-UWA communications, there still exists shortened ISI due to multipath propagation in each sub-channel, joint receive diversity and adaptive equalization is often used as traditional technique to suppress ISI. However, a large receive array used for high diversity gain can lead to complex and expensive receive configuration and hamper the miniaturization of UWA sensor nodes [16,17,18,19,20]. Deficiencies of joint receive diversity and adaptive equalization mentioned above provide the motivation for the work of the paper, that is, removing the ISI in FMT-UWA communications with relatively simple receive configuration.

Space–time block coding (STBC) is an attractive diversity technique that can utilize spatial diversity offered by transmit elements as well as receive elements through mapping the information sequence to multiple transmit elements based on the orthogonal design principle [21,22,23,24,25,26,27,28]. Since STBC can substitute multiple transmit elements for multiple receive elements to offer high diversity gain, it is beneficial to ISI suppression with the simple receive configuration and minimization of UWA sensors. However, STBC is traditionally designed for frequency-flat channels, and the performance can be degraded when it is directly applied to frequency-selective fading channels.

Much research has been carried out to apply the design principle of STBC to frequency-selective fading channels for achieving transmit diversity gain in the last decades, and time-reversal (TR) STBC as an extension technique of STBC has received many research interests [27,28]. As with traditional STBC, TR-STBC is also designed based on the orthogonal design principle, but the encoding is based on symbol blocks instead of individual symbols in traditional STBC. Furthermore, since the spatio-temporal matched filtering (MF) is used in the decoding of TR-STBC, the decoding of TR-STBC can not only decouple symbol blocks but also significantly suppress ISI caused by multipath propagation of frequency-selective fading channels and alleviate the complexity of residual ISI suppression.

In the paper, to eliminate ISI in FMT-UWA communications with simple receive configuration, the joint TR-STBC and adaptive equalization for FMT-UWA communications is proposed in consideration of the merits of TR-STBC. In the proposed method, TR-STBC encoding is used to mapping the information sequences to multiple FMT modulators at the transmitter, TR-STBC decoding is used to decouple multiple symbol blocks and preprocess ISI and adaptive equalization is used as post-processor to deal with residual ISI at the receiver. Since the TR-STBC technique can substitute multiple transmit elements for multiple receive elements to offer high diversity gain and obviously mitigate ISI, and adaptive equalization can further remove residual ISI, the proposed method can obtain high reliability with simple receive configuration.

In the paper, the proposed method is analyzed based on the scenario with two transmit elements and one receive element, but the principle can be extended to FMT-UWA communications with multiple transmit elements and multiple receive elements. The theoretical basis of the proposed method is described in detail, the assessment is performed using simulation analysis, and data is collected in an experiment carried out in a pool with multipath propagation, and the validity is proved by comparing the proposed method with two methods: (1) joint single-input–single-output (SISO) and adaptive equalization where only one transmit element and one receive element are used and no spatial diversity is exploited for diversity gain; (2) joint single-input–multiple-output (SIMO) and adaptive equalization where one transmit element and multiple receive elements are used and receive diversity is adopted for diversity gain. Moreover, it should be noted that MF and adaptive equalization exploited by the proposed method are also used in the two methods mentioned above in order to compare three methods with the same ISI suppression process, that is, the performance differences among three methods are only caused by the number of elements offering spatial diversity gain.

The contribution of the paper is threefold: (1) TR-STBC is first applied to FMT-UWA communications to replace receive diversity with transmit diversity for acquiring high diversity gain to suppress ISI with simple receive configuration; (2) adaptive equalization is exploited to suppress residual ISI after TR-STBC decoding; (3) simulation and the real experiment are performed to verify the proposed method.

This paper is organized as follows. Section 2 describes the transmit structure of the proposed method consisting of the system model, TR-STBC encoding and FMT modulation. Section 3 describes the receiver structure, where FMT demodulation, TR-STBC decoding and adaptive equalization are discussed in detail. Section 4 presents the simulation and experiment results of the proposed method, and compares the proposed method with the other two methods including joint SISO and adaptive equalization and joint SIMO and adaptive equalization. Section 5 summarizes the conclusion.

## 2. Transmit Structure

### 2.1. System Model

Before describing the principle of the proposed method, some notations used in the paper are first introduced. Vectors and matrices are specified by bold lowercase and uppercase, respectively. The operators ${\left(\cdot \right)}^{*}$ and $\bar{\left(\cdot \right)}$ stand for conjugate operation and TR complex conjugate operation, respectively. The operator ∗ denotes convolution. Moreover, all time domain signals in the paper are expressed as discrete form, where the time sample interval is equal to the symbol interval of each FMT sub-band and is omitted in the follow section for concise expression.

In the paper, the proposed method is analyzed based on the scenario with two transmit elements and one receive element for convenient analysis. Certainly, according to the requirement of practical application, the proposed methods can be applied to the communications with multiple transmit elements and multiple receive elements.

**Figure 1:**
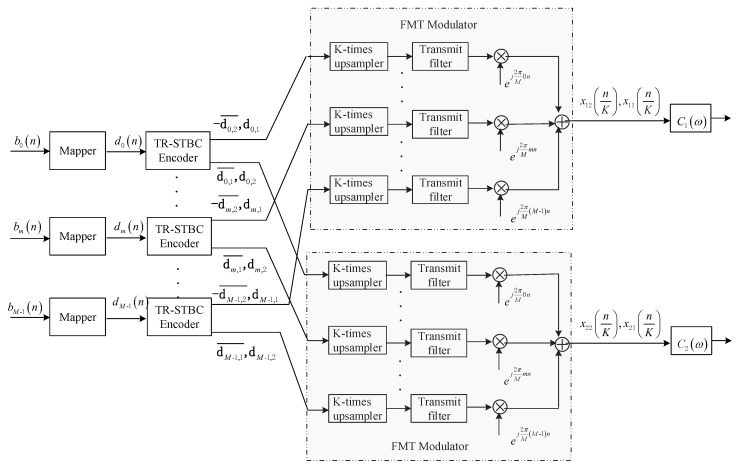
Block diagram for transmit structure.

The base-band equivalent transmit structure of the proposed method is depicted in Figure 1, where the communication band is divided into M sub-bands, and the information sequence on each sub-band is sequentially processed by mapping, TR-STBC encoding and FMT modulating before being transmitted.

### 2.2. Time-Reversal Space-Time Block Coding (TR-STBC) Encoding and Filtered Multitone (FMT) Modulation

At the transmitter, based on the principle of TR-STBC encoding, the mapped sequence on each sub-band $\left\{d_{m}\left(n\right)\right\},m=0,\ldots ,M-1,\;n=0,\ldots ,2N-1$ is firstly divided into two symbol blocks $d_{m,1}$ and $d_{m,2}$, where $d_{m,1}=\left\{d_{m,1}\left(0\right),d_{m,1}\left(1\right),\ldots ,d_{m,1}\left(N-1\right)\right\}$ and $d_{m,2}=\left\{d_{m,2}\left(0\right),d_{m,2}\left(1\right),\ldots ,d_{m,2}\left(N-1\right)\right\}$.

After TR-STBC encoding [27,28], the signal pattern on the *m*-th sub-band can be expressed as:

(1) $$D_{m}=\left[\begin{array}{cc} d_{m,1} & -\bar{d_{m,2}}\\ d_{m,2} & \bar{d_{m,1}} \end{array}\right]$$

where $\bar{d_{m,1}}=\left\{d_{m,1}^{*}\left(N-1\right),d_{m,1}^{*}\left(N-2\right),\ldots ,d_{m,1}^{*}\left(0\right)\right\}$ denotes the TR complex conjugate of $d_{m,1}$, $-\bar{d_{m,2}}=\left\{-d_{m,2}^{*}\left(N-1\right),-d_{m,2}^{*}\left(N-2\right),\ldots ,-d_{m,2}^{*}\left(0\right)\right\}$ denotes the TR complex conjugate and sign inverted version of $d_{m,2}$, and the element at the α-th row and the *s*-th column in $D_{m}$ denotes the symbol block passed to the α-th FMT modulator in the *s*-th time frame.

After FMT modulation, the signals transmitted from two elements can be expressed as:

(2) $$x\left(\frac{n}{K}\right)=\left[\begin{array}{cc} x_{11}\left(\frac{n}{K}\right) & x_{12}^{}\left(\frac{n}{K}\right)\\ x_{21}\left(\frac{n}{K}\right) & x_{22}^{}\left(\frac{n}{K}\right) \end{array}\right]$$

where K denotes the sample multiple of the upsampler in FMT modulator, the element $x_{\alpha s}\left(n/K\right),\alpha =1,2,s=1,2$ denotes the signal transmitted from the α-th element in the *s*-th transmit time frame, and expressions are as follows,

(3) $$\begin{array}{l} x_{11}\left(\frac{n}{K}\right)=\sum_{m=0}^{M-1}\sum_{k=0}^{N-1}d_{m,1}\left(k\right)g_{t}\left(\frac{n}{K}-k\right)e^{j\frac{2\pi }{M}mn}\\ x_{21}\left(\frac{n}{K}\right)=\sum_{m=0}^{M-1}\sum_{k=0}^{N-1}d_{m,2}\left(k\right)g_{t}\left(\frac{n}{K}-k\right)e^{j\frac{2\pi }{M}mn}\\ x_{12}^{}\left(\frac{n}{K}\right)=-\sum_{m=0}^{M-1}\sum_{k=0}^{N-1}d_{m,2}^{*}\left(N-1-k\right)g_{t}\left(\frac{n}{K}-k\right)e^{j\frac{2\pi }{M}mn}\\ x_{22}^{}\left(\frac{n}{K}\right)=\sum_{m=0}^{M-1}\sum_{k=0}^{N-1}d_{m,1}^{*}\left(N-1-k\right)g_{t}\left(\frac{n}{K}-k\right)e^{j\frac{2\pi }{M}mn} \end{array}$$

where $g_{t}\left(n/K\right)$ is the *K*-time upsampling of the time domain response of transmit filter $g_{t}\left(n\right)$ with length $N_{g}$.

## 3. Receive Structure

### 3.1. System Model

Passing through the UWA channels, the received signal in two time frames can be expressed as:

(4) $$\begin{array}{l} r_{1}\left(\frac{n}{K}\right)=\sum_{q=0}^{N_{c}-1}x_{11}\left(\frac{n-q}{K}\right)c_{1}\left(\frac{q}{K}\right)+\sum_{q=0}^{N_{c}-1}x_{21}\left(\frac{n-q}{K}\right)c_{2}\left(\frac{q}{K}\right)+\eta_{1}\left(\frac{n}{K}\right)\\ {r^{\prime }}_{2}\left(\frac{n}{K}\right)=\sum_{q=0}^{N_{c}-1}x_{12}\left(\frac{n-q}{K}\right)c_{1}\left(\frac{q}{K}\right)+\sum_{q=0}^{N_{c}-1}x_{22}\left(\frac{n-q}{K}\right)c_{2}\left(\frac{q}{K}\right)+{\eta^{\prime }}_{2}\left(\frac{n}{K}\right) \end{array}$$

where $c_{\alpha }\left(n/K\right),\;\alpha =1,2$, denotes the channel response between the α-th transmit element and the receive element, $N_{c}$ denotes the length of the channels, $\eta_{1}\left(n/K\right)$ and ${\eta^{\prime }}_{2}\left(n/K\right)$ denote the noises in two time frames.

**Figure 2:**
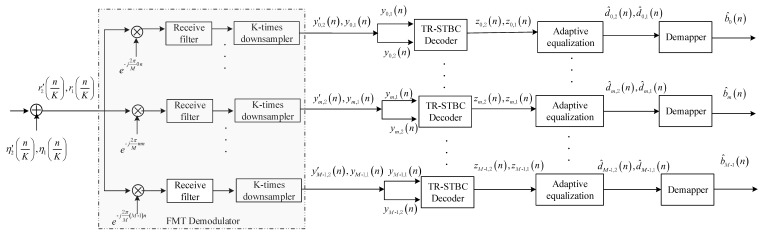
Block diagram for receive structure.

The base-band equivalent receive structure of the proposed method is shown in Figure 2, where the received signal in each time frame is subsequently processed by FMT demodulation, TR-STBC decoding, adaptive equalization, and demapping before acquiring the estimation of each information sequence.

### 3.2. FMT Demodulation

Referring to Figure 2, for the *m*-th sub-band, the demodulated signals in two time frames can be expressed as:

(5) $$\begin{array}{l} y_{m,1}\left(n\right)=\sum_{i=0}^{M-1}\sum_{l=0}^{N-1}f_{\left(i\to m\right)}^{1}\left(l,n\right)d_{i,1}\left(l\right)+\sum_{i=0}^{M-1}\sum_{l=0}^{N-1}f_{\left(i\to m\right)}^{2}\left(l,n\right)d_{i,2}\left(l\right)+w_{m,1}\left(n\right)\\ {y^{\prime }}_{m,2}\left(n\right)=-\sum_{i=0}^{M-1}\sum_{l=0}^{N-1}f_{\left(i\to m\right)}^{1}\left(l,n\right)d_{i,2}^{*}\left(N-1-l\right)+\sum_{i=0}^{M-1}\sum_{l=0}^{N-1}f_{\left(i\to m\right)}^{2}\left(l,n\right)d_{i,1}^{*}\left(N-1-l\right)+{w^{\prime }}_{m,2}\left(n\right) \end{array}$$

where $f_{\left(i\to m\right)}^{\alpha }\left(l,n\right),\;m=0,\ldots ,\;M-1,\;i=0,\ldots ,\;M-1,\;\alpha =1,\;2$, denotes the time domain response of the composite channel from the i-th upsampler of the α-th FMT modulator to the m-th downsampler of the FMT demodulator, $w_{m,1}\left(n\right)$ and ${w^{\prime }}_{m,2}\left(n\right)$ denote the output noises of the FMT demodulator in two time frames. The expressions of $f_{\left(i\to m\right)}^{\alpha }\left(l,n\right)$, $w_{m,1}\left(n\right)$ and ${w^{\prime }}_{m,2}\left(n\right)$ are as follows,

(6) $$\begin{array}{l} f_{\left(i\to m\right)}^{1}\left(l,n\right)=\sum_{k=0}^{N_{g}-1}\sum_{q=0}^{N_{c}-1}g_{t}\left(\frac{k-q}{K}-l\right)c_{1}\left(\frac{q}{K}\right)e^{-j\frac{2\pi }{M}iq}g_{r}\left(n-\frac{k}{K}\right)e^{j\frac{2\pi }{M}\left(i-m\right)k}\\ f_{\left(i\to m\right)}^{2}\left(l,n\right)=\sum_{k=0}^{N_{g}-1}\sum_{q=0}^{N_{c}-1}g_{t}\left(\frac{k-q}{K}-l\right)c_{2}\left(\frac{q}{K}\right)e^{-j\frac{2\pi }{M}iq}g_{r}\left(n-\frac{k}{K}\right)e^{j\frac{2\pi }{M}\left(i-m\right)k}\\ w_{m,1}\left(n\right)=\sum_{k=0}^{N-1}\eta_{1}\left(\frac{k}{K}\right)g_{r}\left(n-\frac{k}{K}\right)e^{-j\frac{2\pi }{M}mk}\\ {w^{\prime }}_{m,2}\left(n\right)=\sum_{k=0}^{N-1}{\eta^{\prime }}_{2}\left(\frac{k}{K}\right)g_{r}\left(n-\frac{k}{K}\right)e^{-j\frac{2\pi }{M}mk} \end{array}$$

where $g_{r}\left(n\right)$ denotes the time response of the receive filter matched to the transmit filter $g_{t}\left(n\right)$, i.e., the frequency responses of the transmit filter and the receive filter satisfy with $G_{r}\left(\omega \right)=G_{t}^{*}\left(\omega \right)$.

Equation (5) can be further expressed as:

(7) $$\begin{array}{cc} y_{m,1}\left(n\right) & =\sum_{l=0}^{N-1}f_{\left(m\to m\right)}^{1}\left(l,n\right)d_{m,1}\left(l\right)+\sum_{\begin{array}{l} i=0\\ i\neq m \end{array}}^{M-1}\sum_{l=0}^{N-1}f_{\left(i\to m\right)}^{1}\left(l,n\right)d_{i,1}\left(l\right)\\ & +\sum_{l=0}^{N-1}f_{\left(m\to m\right)}^{2}\left(l,n\right)d_{m,2}\left(l\right)+\sum_{\begin{array}{l} i=0\\ i\neq m \end{array}}^{M-1}\sum_{l=0}^{N-1}f_{\left(i\to m\right)}^{2}\left(l,n\right)d_{i,2}\left(l\right)+w_{m,1}\left(n\right)\\ {y^{\prime }}_{m,2}\left(n\right) & =-\left[\sum_{l=0}^{N-1}f_{\left(m\to m\right)}^{1}\left(l,n\right)d_{m,2}^{*}\left(N-1-l\right)+\sum_{\begin{array}{l} i=0\\ i\neq m \end{array}}^{M-1}\sum_{l=0}^{N-1}f_{\left(i\to m\right)}^{1}\left(l,n\right)d_{i,2}^{*}\left(N-1-l\right)\right]\\ & +\sum_{l=0}^{N-1}f_{\left(m\to m\right)}^{2}\left(l,n\right)d_{m,1}^{*}\left(N-1-l\right)+\sum_{\begin{array}{l} i=0\\ i\neq m \end{array}}^{M-1}\sum_{l=0}^{N-1}f_{\left(i\to m\right)}^{2}\left(l,n\right)d_{i,1}^{*}\left(N-1-l\right)+{w^{\prime }}_{m,2}\left(n\right) \end{array}$$

Equation (7) indicates that the ICI are included in the demodulated signals. In FMT, the ICI is a minor concern due to separated wide sub-bands and high spectral containment, and the effect can be neglected even when the Doppler spread caused by the water motion exists. In the paper, the concerned focus is exploiting joint TR-STBC and adaptive equalization to eliminate ISI in FMT-UWA communications, no account is taken of the ICI because of the negligibly slight effect. When the ICI is neglected, Equation (7) can be simplified as:

(8) $$\begin{array}{l} y_{m,1}\left(n\right)=\sum_{l=0}^{N-1}f_{\left(m\to m\right)}^{1}\left(l,n\right)d_{m,1}\left(l\right)+\sum_{l=0}^{N-1}f_{\left(m\to m\right)}^{2}\left(l,n\right)d_{m,2}\left(l\right)+w_{m,1}\left(n\right)\\ {y^{\prime }}_{m,2}\left(n\right)=-\sum_{l=0}^{N-1}f_{\left(m\to m\right)}^{1}\left(l,n\right)d_{m,2}^{*}\left(N-1-l\right)+\sum_{l=0}^{N-1}f_{\left(m\to m\right)}^{2}\left(l,n\right)d_{m,1}^{*}\left(N-1-l\right)+{w^{\prime }}_{m,2}\left(n\right) \end{array}$$

### 3.3. TR-STBC Decoding and Adaptive Equalization

Before TR-STBC decoding, the demodulated signal in the second time frame ${y^{\prime }}_{m,2}\left(n\right)$ is firstly processed by time reversed and complex conjugated operation,

(9) $$\begin{array}{l} y_{m,2}\left(n\right)=\bar{{y^{\prime }}_{m,2}\left(n\right)}=-\sum_{l=0}^{N-1}\bar{f_{\left(m\to m\right)}^{1}\left(l,n\right)}d_{m,2}^{}\left(l\right)+\sum_{l=0}^{N-1}\bar{f_{\left(m\to m\right)}^{2}\left(l,n\right)}d_{m,1}^{}\left(l\right)+w_{m,2}\left(n\right)\\ w_{m,2}\left(n\right)=\bar{{w^{\prime }}_{m,2}\left(n\right)}={w^{\prime }}_{m,2}^{*}\left(N-1-n\right) \end{array}$$

The signals $y_{m,1}\left(n\right)$ and $y_{m,2}\left(n\right)$ be inputted to the m-th TR-STBC decoder can be expressed as:

(10) $$\left[\begin{array}{c} y_{m,1}\left(n\right)\\ y_{m,2}\left(n\right) \end{array}\right]=\left[\begin{array}{cc} \sum_{l=0}^{N-1}f_{\left(m\to m\right)}^{1}\left(l,n\right) & \sum_{l=0}^{N-1}f_{\left(m\to m\right)}^{2}\left(l,n\right)\\ \sum_{l=0}^{N-1}\bar{f_{\left(m\to m\right)}^{2}\left(l,n\right)} & -\sum_{l=0}^{N-1}\bar{f_{\left(m\to m\right)}^{1}\left(l,n\right)} \end{array}\right]\left[\begin{array}{c} d_{m,1}^{}\left(l\right)\\ d_{m,2}^{}\left(l\right) \end{array}\right]+\left[\begin{array}{c} w_{m,1}\left(n\right)\\ w_{m,2}\left(n\right) \end{array}\right]$$

The corresponding frequency domain form of Equation (10) is:

(11) $$\underset{Y_{m}\left(\omega \right)}{\underset{︸}{\left[\begin{array}{c} Y_{m,1}\left(\omega \right)\\ Y_{m,2}\left(\omega \right) \end{array}\right]}}=\underset{F_{\left(m,m\right)}\left(\omega \right)}{\underset{︸}{\left[\begin{array}{cc} F_{\left(m\to m\right)}^{1}\left(\omega \right) & F_{\left(m\to m\right)}^{2}\left(\omega \right)\\ {\left(F_{\left(m\to m\right)}^{2}\left(\omega \right)\right)}^{*} & -{\left(F_{\left(m\to m\right)}^{1}\left(\omega \right)\right)}^{*} \end{array}\right]}}\underset{D_{m}\left(\omega \right)}{\underset{︸}{\left[\begin{array}{c} D_{m,1}^{}\left(\omega \right)\\ D_{m,2}^{}\left(\omega \right) \end{array}\right]}}+\underset{W_{m}\left(\omega \right)}{\underset{︸}{\left[\begin{array}{c} W_{m,1}\left(\omega \right)\\ W_{m,2}\left(\omega \right) \end{array}\right]}}$$

where

(12) $$\begin{array}{l} F_{\left(m\to m\right)}^{\alpha }\left(\omega \right)=G_{t}\left(\omega \right)C_{\alpha ,m}\left(\omega \right)G_{t}^{*}\left(\omega \right)\;\alpha =1,2\\ {\left(F_{\left(m\to m\right)}^{\alpha }\left(\omega \right)\right)}^{*}=G_{t}\left(\omega \right)C_{\alpha ,m}^{*}\left(\omega \right)G_{t}^{*}\left(\omega \right)\;\alpha =1,2\\ C_{\alpha ,m}\left(\omega \right)=C_{\alpha }\left(\omega +\frac{2\pi }{M}m\right)\;\alpha =1,2\\ W_{m,1}\left(\omega \right)=\eta_{1}\left(\omega -\frac{2\pi }{M}m\right)G_{t}^{*}\left(\omega \right)\\ W_{m,2}\left(\omega \right)=\eta_{2}^{*}\left(\omega -\frac{2\pi }{M}m\right)G_{t}^{}\left(\omega \right) \end{array}$$

In Equation (12), $C_{\alpha }\left(\omega \right),\alpha =1,2$ denotes the frequency domain response of the channel between the α-th transmit element and the receive element, $C_{\alpha ,m}\left(\omega \right),\alpha =1,2,m=0,\ldots ,M-1$ denotes the frequency domain response of the *m*-th sub-channel between the α-th transmit element and the receive element, $\eta_{1}\left(\omega \right)$ and $\eta_{2}^{*}\left(\omega \right)$ denote the frequency domain form of channel noises shown in Equation (4).

Based on Equations (11) and (12), the composite channel response matrix $F_{\left(m\to m\right)}\left(\omega \right)$ can be further expressed as:

(13) $$F_{\left(m\to m\right)}\left(\omega \right)=G_{t}\left(\omega \right)\underset{c_{m}\left(\omega \right)}{\underset{︸}{\left[\begin{array}{cc} C_{1,m}\left(\omega \right) & C_{2,m}\left(\omega \right)\\ C_{2,m}^{*}\left(\omega \right) & -C_{1,m}^{*}\left(\omega \right) \end{array}\right]}}G_{t}^{*}\left(\omega \right)$$

where the channel response matrix $C_{m}\left(\omega \right)$ is orthogonal,

(14) $$\begin{array}{cc} C_{m}^{H}\left(\omega \right)C_{m}\left(\omega \right) & =\left[\begin{array}{cc} C_{1,m}^{*}\left(\omega \right) & C_{2,m}\left(\omega \right)\\ C_{2,m}^{*}\left(\omega \right) & -C_{1,m}\left(\omega \right) \end{array}\right]\left[\begin{array}{cc} C_{1,m}\left(\omega \right) & C_{2,m}\left(\omega \right)\\ C_{2,m}^{*}\left(\omega \right) & -C_{1,m}^{*}\left(\omega \right) \end{array}\right]\\ & =\left({\left|C_{1,m}\left(\omega \right)\right|}^{2}+{\left|C_{2,m}\left(\omega \right)\right|}^{2}\right)\left[\begin{array}{cc} 1 & 0\\ 0 & 1 \end{array}\right] \end{array}$$

Assuming each sub-channel response can be estimated without error, the complex conjugate transposed matrix $C_{m}^{H}\left(\omega \right)$ can be used to filter the demodulated signal vector $Y_{m}\left(\omega \right)$ in TR-STBC decoding. After TR-STBC decoding, the *m*-th output signal vector can be expressed as:

(15) $$\underset{Z_{m}\left(\omega \right)}{\underset{︸}{\left[\begin{array}{c} Z_{m,1}\left(\omega \right)\\ Z_{m,2}\left(\omega \right) \end{array}\right]}}=\underset{H_{m}\left(\omega \right)}{\underset{︸}{{\left|G_{t}\left(\omega \right)\right|}^{2}\left({\left|C_{1,m}\left(\omega \right)\right|}^{2}+{\left|C_{2,m}\left(\omega \right)\right|}^{2}\right)}}\underset{D_{m}^{}\left(\omega \right)}{\underset{︸}{\left[\begin{array}{c} D_{m,1}^{}\left(\omega \right)\\ D_{m,2}^{}\left(\omega \right) \end{array}\right]}}+\underset{\xi_{m}\left(\omega \right)}{\underset{︸}{\left[\begin{array}{c} \xi_{m,1}\left(\omega \right)\\ \xi_{m,2}\left(\omega \right) \end{array}\right]}}$$

where $H_{m}\left(\omega \right)$ denotes the frequency domain response of the *m*-th composite channel after TR-STBC decoding, $\xi_{m}\left(\omega \right)$ denotes the frequency domain form of the noise vector,

(16) $$\left[\begin{array}{c} \xi_{m,1}\left(\omega \right)\\ \xi_{m,2}\left(\omega \right) \end{array}\right]=\left[\begin{array}{c} C_{1,m}^{*}\left(\omega \right)W_{m,1}\left(\omega \right)+C_{2,m}\left(\omega \right)W_{m,2}\left(\omega \right)\\ C_{2,m}^{*}\left(\omega \right)W_{m,1}\left(\omega \right)-C_{1,m}\left(\omega \right)W_{m,2}\left(\omega \right) \end{array}\right]$$

The corresponding time domain form of Equation (15) can be expressed as:

(17) $$\begin{array}{cc} \underset{z_{m}\left(n\right)}{\underset{︸}{\left[\begin{array}{c} z_{m,1}\left(n\right)\\ z_{m,2}\left(n\right) \end{array}\right]}} & =\sum_{l=0}^{N-1}h_{m}\left(l,n\right)\underset{d_{m}^{}\left(l\right)}{\underset{︸}{\left[\begin{array}{c} d_{m,1}^{}\left(l\right)\\ d_{m,2}^{}\left(l\right) \end{array}\right]}}+\underset{\xi_{m}\left(n\right)}{\underset{︸}{\left[\begin{array}{c} \xi_{m,1}\left(n\right)\\ \xi_{m,2}\left(n\right) \end{array}\right]}}\\ & =\underset{desired\;symbols}{\underset{︸}{h_{m}\left(n,n\right)\left[\begin{array}{c} d_{m,1}^{}\left(n\right)\\ d_{m,2}^{}\left(n\right) \end{array}\right]}}+\underset{residual\;ISI}{\underset{︸}{\sum_{\begin{array}{l} l=0\\ l\neq n \end{array}}^{N-1}h_{m}\left(l,n\right)\left[\begin{array}{c} d_{m,1}^{}\left(l\right)\\ d_{m,2}^{}\left(l\right) \end{array}\right]}}+\underset{noise}{\underset{︸}{\left[\begin{array}{c} \xi_{m,1}\left(n\right)\\ \xi_{m,2}\left(n\right) \end{array}\right]}} \end{array}$$

where

(18) $$\begin{array}{l} h_{m}\left(l,n\right)=\sum_{\alpha =1}^{2}\sum_{\lambda =0}^{N_{g}-1}\sum_{k=0}^{N_{g}-1}\sum_{q=0}^{N_{c}-1}g\left(\frac{k-q}{K}-l\right)c_{\alpha ,m}\left(\frac{q}{K}\right)g_{r}\left(\lambda -\frac{k}{K}\right)\bar{c_{\alpha ,m}\left(n-\lambda \right)}\\ c_{\alpha ,m}\left(\frac{q}{K}\right)=c_{\alpha }\left(\frac{q}{K}\right)e^{-j\frac{2\pi }{M}mq}\;\alpha =1,2\\ \xi_{m,1}\left(n\right)=\sum_{\lambda =0}^{N-1}w_{m,1}\left(\lambda \right)\bar{c_{1,m}\left(n-\lambda \right)}+\sum_{\lambda =0}^{N-1}w_{m,2}\left(\lambda \right)c_{2,m}\left(n-\lambda \right)\\ \xi_{m,2}\left(n\right)=\sum_{\lambda =0}^{N-1}w_{m,1}\left(\lambda \right)\bar{c_{2,m}\left(n-\lambda \right)}-\sum_{\lambda =0}^{N-1}w_{m,2}\left(\lambda \right)c_{1,m}\left(n-\lambda \right)\\ \bar{c_{\alpha ,m}\left(n\right)}=c_{\alpha ,m}^{*}\left(N_{c}-1-n\right)\;\alpha =1,2 \end{array}$$

Equation (17) indicates that two symbol blocks are completely decoupled and transmit diversity has been achieved after TR-STBC decoding. Furthermore, it can also be observed from Equation (17) that three terms are contained in the signals after TR-STBC decoding; the first term is the desired symbols, the second term is the residual ISI, and the third term is the noise. As stated previously, the concerned focus of the paper is ISI suppression, and noise is not of interest here and the effect can be ignored when the transmitted power is sufficient.

To eliminate the residual ISI and correctly recover information symbols, multiple adaptive equalizers are used as post-processor after TR-STBC decoding. In the proposed method, linear equalizer (LE) is used for avoiding error propagation, and the output signal of the *m*-th LE can be expressed as:

(19) $$\left[\begin{array}{c} {\hat{d}}_{m,1}\left(n\right)\\ {\hat{d}}_{m,2}\left(n\right) \end{array}\right]=\sum_{\gamma =-N_{1}}^{N_{2}}e_{m}\left(\gamma \right)\left[\begin{array}{c} z_{m,1}^{}\left(n-\gamma \right)\\ z_{m,2}^{}\left(n-\gamma \right) \end{array}\right]$$

where $e_{m}\left(\gamma \right)$ denotes the γ-th coefficient of the *m*-th LE with length $N_{eq}=N_{1}+N_{2}+1$. In the proposed method, the recursive least square (RLS) algorithm is used to adjust the equalization coefficients due to the fast convergence.

## 4. Performance Assessment

### 4.1. Two Methods for Comparison

**Figure 3:**
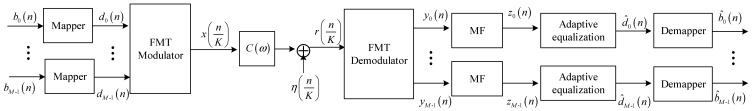
Block diagram for joint single-input–single-output (SISO) and adaptive equalization.

**Figure 4:**
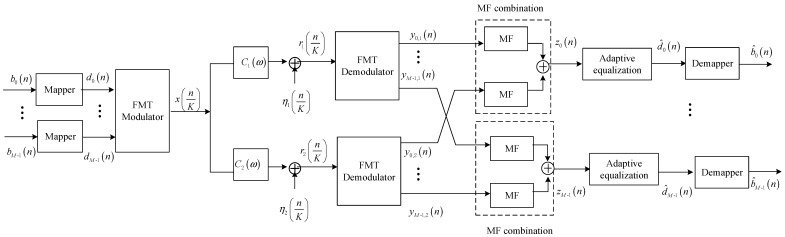
Block diagram for joint single-input–multiple-output (SIMO) and adaptive equalization.

In this section, the performance of the proposed method is assessed using simulation analysis and data collected in a real experiment carried out in a pool with multipath propagation. For performance comparison, the two methods, i.e., the joint SISO with adaptive equalization method and the joint SIMO with adaptive equalization, are analyzed and realized in the same simulation and experimental conditions. The base-band equivalent system structures of two methods for comparison are shown in Figure 3 and Figure 4, where the structures of FMT modulator and demodulator are shown in Figure 1 and Figure 2. Referring to Figure 3 and Figure 4, it can be observed that MF used in the TR-STBC decoding is also exploited in the two methods in order that three methods can be compared when the same ISI suppression techniques are adopted. Moreover, for comparing the ISI suppression performance of transmit diversity offered by TR-STBC with that of receive diversity offered by SIMO, joint SIMO and adaptive equalization is analyzed based on one transmit element and two receive elements. In the following section, for simplicity of expression, joint SISO with adaptive equalization is abbreviated as the SIMO method, and joint SIMO with adaptive equalization is abbreviated as the SIMO method.

### 4.2. Simulation

#### 4.2.1. Channel Model

**Figure 5:**
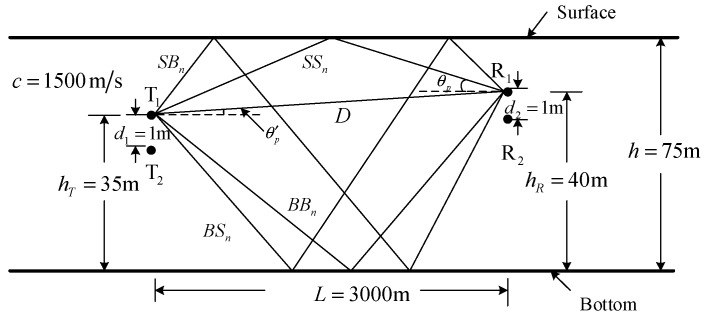
Simulation channel setup.

The channel model for simulation is constructed based on the shallow water multipath geometry with a certain number of propagation paths taken into account [29,30]. The simulation setup is shown in Figure 5.

Referring to Figure 5, the communication distance is L=3000 m, the sound speed is c=1500 m/s, the depth of water is h=75 m, the bottom was flat, the transmit array and the receive array both have two elements spaced by 1 m, the first transmit element is deployed at 35 m above the bottom, and the first receive element is placed at 40 m above the bottom. Five types of propagation path are considered in Figure 5, where D denotes the direct path, $SB_{n}$ denotes the path that the first reflection is the surface reflection and the last reflection is the bottom reflection, n denotes the number of bottom reflections, the meanings of $SS_{n}$, $BS_{n}$ and $BB_{n}$ are similar to that of $SB_{n}$. Each propagation path is characterized by the path gain, time delay and arrival angle, which can be computed from the length of propagation path. The length of each path in Figure 5 can be computed as $l_{p}=\sqrt{L^{2}+H^{2}}$, the arrival angle can be computed as $\theta_{p}=K\tan^{-1}\left(H/L\right)$, where,

(20) $$\begin{array}{lll} H=h_{R}-h_{T}, & K=-1, & for\;path\;D\\ H=2nh-h_{R}-h_{T}, & K=1, & for\;path\;SS_{n}\\ H=2nh-h_{T}+h_{R}, & K=-1, & for\;path\;SB_{n}\\ H=2nh+h_{T}-h_{R}, & K=1, & for\;path\;BS_{n}\\ H=2\left(n-1\right)h+h_{T}+h_{R}, & K=-1, & for\;path\;BB_{n} \end{array}$$

The time delay can be computed as $\tau_{p}=l_{p}/c$. The path gain $c_{p}$ is characterized by the magnitude $\left|c_{p}\right|$ and the phase $∠c_{p}$. The phase $∠c_{p}$ is computed as $∠c_{p}=-2\pi f_{c}\tau_{p}$, where $f_{c}$ is carrier frequency. The magnitude $\left|c_{p}\right|$ is computed as $\left|c_{p}\right|=\Gamma_{p}/\sqrt{A\left(l_{p}\right)}$, where $\Gamma_{p}$ is boundary reflection loss and $A\left(l_{p}\right)$ is propagation loss. The boundary reflection loss is computed as $\Gamma_{p}={\left|v_{s}\right|}^{n_{s}}{\left|v_{b}\right|}^{n_{b}}$, where $\left|v_{s}\right|$ and $\left|v_{b}\right|$ denote the magnitudes of surface and bottom reflection coefficients, and $n_{s}$ and $n_{b}$ denote the number of surface and bottom reflections. The propagation loss is computed as $A\left(l_{p}\right)=l_{p}^{k}{\left[a\left(f_{c}\right)\right]}^{l_{p}}$, where k is the spreading factor, $a\left(f_{c}\right)$ denotes absorption loss and is computed as $10\log a\left(f_{c}\right)=0.11f_{c}^{2}/\left(1+f_{c}^{2}\right)+44f_{c}^{2}\left(4100+f_{c}^{2}\right)+2.75\times 10^{-4}f_{c}^{2}+0.0003$ (dB/km) for $f_{c}$ given in kHz. Referring to Figure 5, there is a phase delay $\varphi_{p}=2\pi \left(f_{c}d_{2}/c\right)\sin\theta_{p}$ between the receive elements spaced by $d_{2}$, there is a phase delay ${\varphi^{\prime }}_{p}=2\pi \left(f_{c}d_{1}/c\right)\sin{\theta^{\prime }}_{p}$ between the transmit elements spaced by $d_{1}$, where ${\theta^{\prime }}_{p}=-\theta_{p}$ when the sound speed keeps constant. Therefore, the frequency domain channel response from the first transmit element to each receive element can be expressed as:

(21) $$C_{1,\beta }\left(\omega \right)=\sum_{p=0}^{P-1}c_{p}e^{-j\left(\beta -1\right)\varphi_{p}}e^{-j\omega \tau_{p}},\beta =1,2$$

and the frequency domain channel response from the each transmit element to the first receive element can be expressed as:

(22) $$C_{\alpha ,1}\left(\omega \right)=\sum_{p=0}^{P-1}c_{p}e^{-j\left(\alpha -1\right){\varphi^{\prime }}_{p}}e^{-j\omega \tau_{p}},\alpha =1,2$$

In simulation, each surface reflection is assumed to be lossless, each bottom reflection is set to introduce a 0.75 loss in magnitude, the spread factor is set to k=2, and the number of bottom reflections is set to n=2. The other parameters of the proposed method are shown in Table 1, where the parameters of SISO method and SIMO method are also listed for comparison. From Table 1, it can be observed that the parameters of three methods are the same except for the number of the transmit elements and receive elements.

#### 4.2.2. Simulation Results

Based on the shallow water channel model, simulation results are provided to assess the advantages of the proposed method over SISO method and SIMO method. In the simulation of the proposed method, two transmit elements and the first receive element are used for communication. In the simulation of the SIMO method, the first transmit element and two receive elements are used for communication. In the simulation of the SISO method, the first transmit element and the first receive element are used for communication.

**Figure 6:**
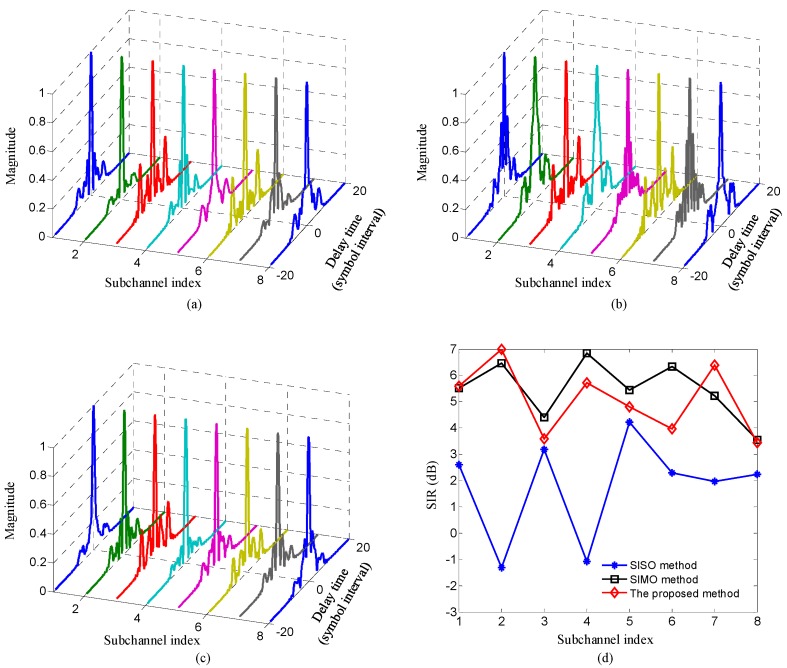
Comparison of the time domain responses and signal-to-interference ratios (SIRs) of composite sub-channels among three methods: (**a**) sub-channel responses of the proposed method; (**b**) sub-channel responses of SISO method; (**c**) sub-channel responses of SIMO method; (**d**) SIRs of three methods.

Figure 6 shows the comparison of time domain responses and signal-to-interference ratios (SIRs) of composite channels between the mapped information sequences $\left\{b_{m}\left(n\right)\right\},m=0,\ldots ,M-1$ and the demodulated sequences $\left\{z_{m}\left(n\right)\right\},m=0,\ldots ,M-1$ among three methods. The SIRs of three methods can be computed as $SIR_{o}={\left|h_{m}\left(n,n\right)\right|}^{2}/\sum_{l=0,l\neq n}^{N-1}{\left|h_{m}\left(l,n\right)\right|}^{2}$, where $h_{m}\left(l,n\right)$ denotes the time domain response of the m-th composite channels, and higher value means less ISI and better ISI suppression performance.

Two observations can be made from Figure 6. Firstly, for each composite sub-channel, owing to the fact that the transmit diversity obtained by the proposed method can further suppress ISI, the magnitude of ISI is reduced and the SIR are improved using the proposed method compared with the SISO method. Secondly, for each sub-channel, due to that the transmit diversity obtained by the proposed method and the receive diversity acquired by the SIMO method leads to similar suppress ISI performance, the magnitude of ISI and the SIR of the proposed method are similar to that of the SIMO method. Moreover, it must be mentioned that since the responses of UWA channels between different transmit elements and receive elements are frequency-selective-fading channels and not the same, the SIRs of the two methods are similar and not equal when the number of elements providing spatial diversity in the proposed method is equal to that in the SIMO method.

**Figure 7:**
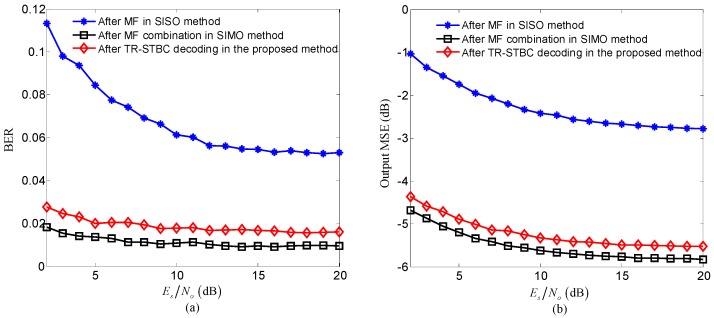
Comparison of the performance after three processing scenarios consisting of time-reversal space-time block coding (TR-STBC) decoding in the proposed method, matched filtering (MF) in the SISO method and MF combination in the SIMO method: (**a**) bit error rate (BER); (**b**) output mean square error (MSE).

The performance after TR-STBC decoding in the proposed method on different signal-to-noise ratio (SNR) $E_{s}/N_{o}$ is shown in Figure 7, where the performance after MF in the SISO method and that after MF combination in the SISO method are also displayed for comparison. In Figure 7, $E_{s}$ denotes the energy of per symbol transmitted on each sub-band, $N_{o}$ denotes the power spectral density of channel noise, bit error rate (BER) and the output mean square error (MSE) computed by the mean square difference between the transmit symbol and the estimate of that symbol are used as performance indicators; lower values of BER and MSE stand for higher performance.

Three observations can be made from Figure 7. Firstly, the performance after TR-STBC decoding in the proposed method is obviously better than that after MF in the SISO method due to better ISI suppression of the proposed method. Secondly, the performance after TR-STBC decoding in the proposed method is similar to that after MF combination in the SIMO method because of closely ISI suppression performance obtained by the proposed method and the SIMO method. Moreover, since the proposed method substitutes transmit diversity for receive diversity used in the SIMO method to maintain high ISI suppress performance, the proposed method can achieve similar performance with more simpler receive configuration and be benefit to the miniaturization of sensor nodes. Thirdly, owing to that the performance is mainly determined by the residual ISI after TR-STBC decoding under high SNR, the performance after TR-STBC decoding in the proposed method tends toward saturation with the increasing of $E_{s}/N_{0}$.

**Figure 8:**
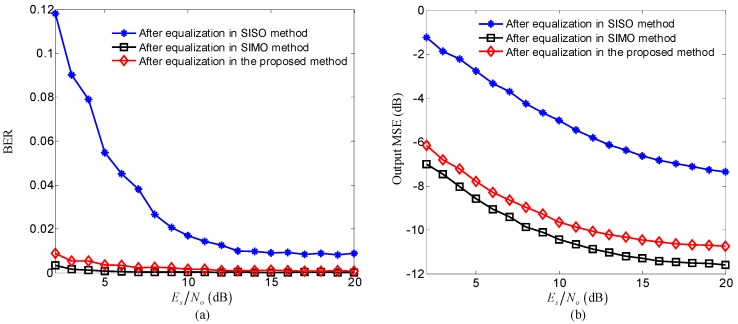
Comparison of the performance after equalization among three methods including of the proposed method, SISO method and SIMO method: (**a**) BER; (**b**) output MSE.

To further suppress residual ISI after TR-STBC decoding, adaptive equalization is used as post-processor in the proposed method. The performance after equalization in the proposed method is shown in Figure 8, where the performance after equalization in SISO method and that after equalization in SIMO method are also displayed for comparison.

Three observations can be made from Figure 8. Firstly, for the proposed method, the performance is further improved after adaptive equalization compared with after TR-STBC decoding due to further ISI suppression. Secondly, since the degree of residual ISI after TR-STBC decoding in the proposed method is similar to that after MF combination in the SIMO method, the performance after equalization in the proposed method is closed to that after MF combination in the SIMO method. Thirdly, the performance after equalization in the proposed method is superior to that after MF in the SISO method due to better ISI suppression performance after TR-STBC decoding in the proposed method.

### 4.3. Experiment

#### 4.3.1. Experiment Setup

For assessing the proposed method in real underwater environment, the experiment was carried out in an indoor pool with four sides covered with acoustic absorbent and the bottom covered with sand. The length, width and depth of the experimental pool were 45 m, 6 m, and 5 m, respectively. Two hemispherical transducers vertically deployed at 2 m and 3 m below the surface were used as transmit sensors, two spherical hydrophone placed at 2 m and 2.5 m below the surface were used as receive sensors, and the communication distance was 8.2 m. The experiments of the SISO method and the SIMO method were carried out in the same conditions for comparison. In the experiment, two transducers and the hydrophone placed at 2.5 m below the surface were used for communication. In the experiment of the SIMO method, the transducer placed at 2 m below the surface and two hydrophones were used for communication. In the experiment of the SISO method, the transducer placed at 2 m below the surface and the hydrophone placed at 2.5 m below the surface were used for communication.

**Figure 9:**
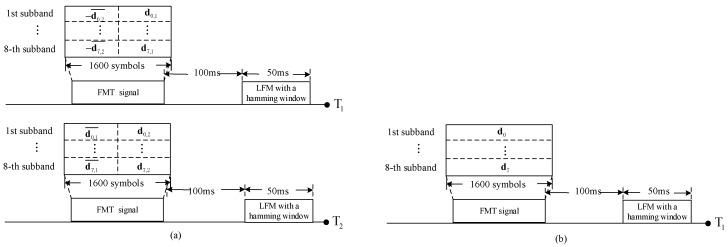
Diagram for transmit signals of three methods: (**a**) the proposed method; (**b**) SISO method and SIMO method.

The transmit signal structure of the proposed method is shown in Figure 9 and that of the SISO method and the SIMO method is also displayed. Referring to Figure 9, the signal transmitted by each transducer is composed by a 50 ms, 8–16kHz linear frequency modulation (LFM) with a hamming window, a 100 ms guard time interval and a FMT signal. The FMT signals of the proposed method can be obtained by modulating the TR-STBC encoded symbols, the FMT signals of the SISO method and the SIMO method can be obtained by modulating the mapped symbols directly. In experiment, the parameters are the same as which in Table 1 except that the number of equalization coefficients on each sub-band is 25.

#### 4.3.2. Experiment Results

**Figure 10:**
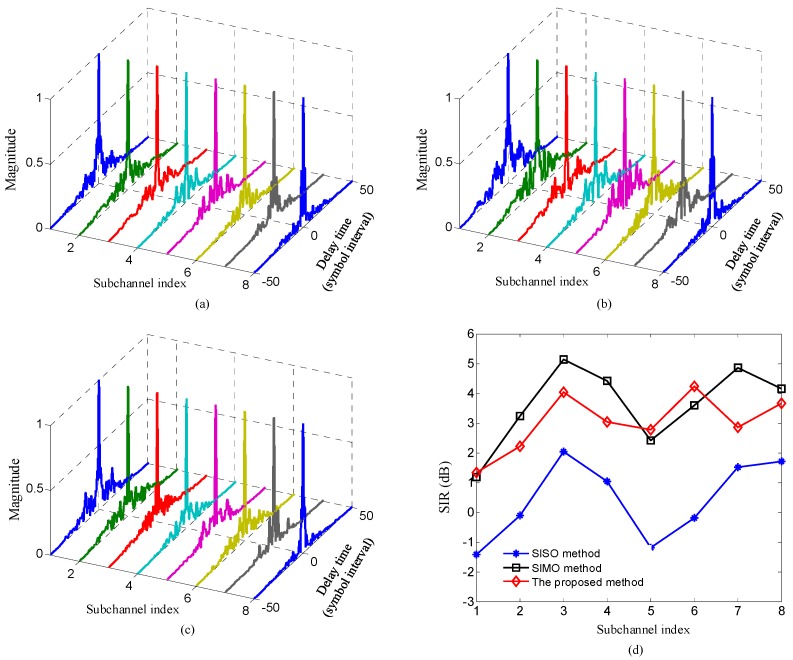
Comparison of the time domain responses and SIRs of composite channels in experiment among three methods: (**a**) sub-channel responses of the proposed method; (**b**) sub-channel responses of the SISO method; (**c**) sub-channel responses of the SIMO method; (**d**) SIRs of three methods.

In the experiment, the sub-channel responses were estimated using recursive least square (RLS) algorithm when the input SNR of the receiver was 20.1dB. Figure 10 compares the time domain responses and SIRs of composite channels among three methods. Figure 10 indicates that for each sub-channel, the further suppressed ISI and improved SIRs can be obtained using the proposed method compared with the SISO method, the similar ISI suppression and SIRs can be acquired using the proposed method compared with the SIMO method. All results shown in Figure 10 are consistent with the corresponding simulation results in Figure 6.

**Figure 11:**
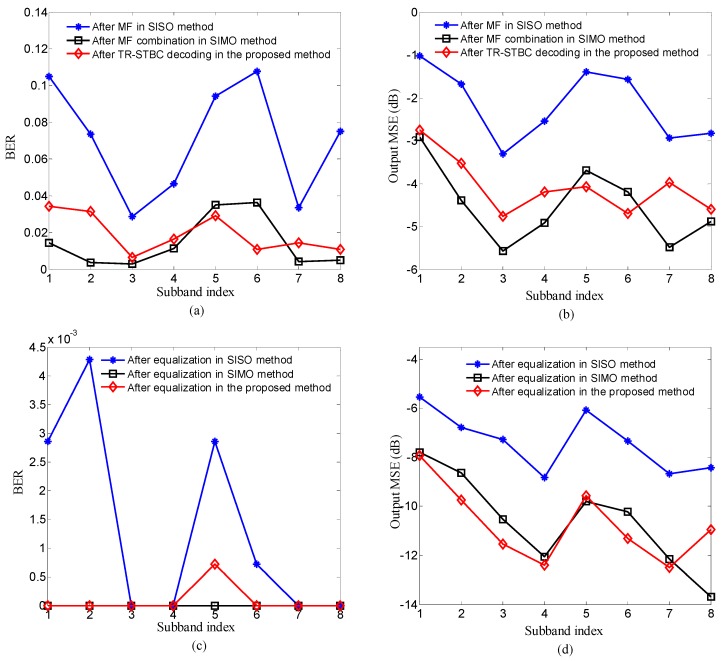
Comparison of the performance for each sub-band among three methods: (**a**) and (**b**) comparing the BER and output MSE after three processing scenarios consisting of TR-STBC decoding in the proposed method, MF in the SISO method and MF combination in the SIMO method; (**c**) and (**d**) comparing the BER and MMSE after adaptive equalization among the three methods.

**Figure 12:**
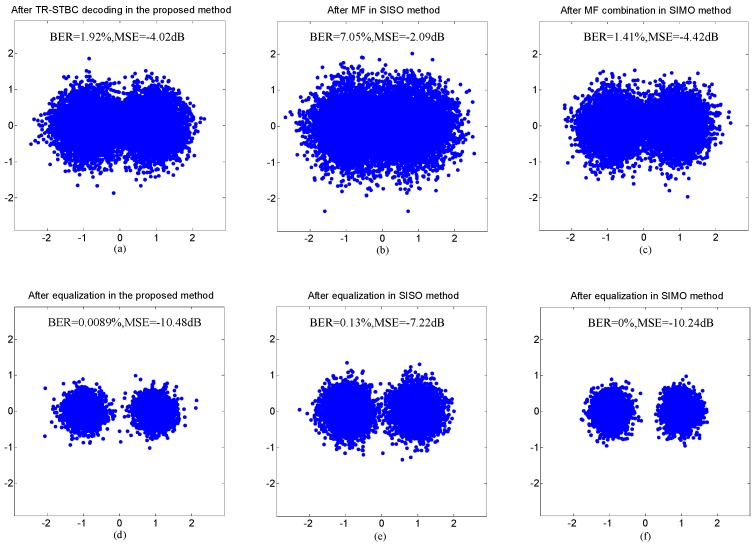
Comparison of total performance among the three communication methods in the experiment: (**a**) and (**d**) after TR-STBC decoding and adaptive equalization in the proposed method; (**b**) and (**e**) after MF and adaptive equalization in the SISO method; (**c**) and (**f**) after MF combination and adaptive equalization in the SIMO method.

In Figure 11, for each sub-band, the performance after TR-STBC decoding in the proposed method was compared with that after two processing scenarios consisting of MF in the SISO method and MF combination in the SIMO method, and the performance after equalization was also compared among three methods. The comparison of total performance among three methods is shown in Figure 12, where the BER and output MSE of each method are the average over all sub-bands and the dots in scatterplots correspond to all information symbols transmitted on all sub-bands.

Referring to Figure 11 and Figure 12, it can be observed that the performance after TR-STBC decoding and adaptive equalization in the proposed method is superior to that after MF and adaptive equalization in the SISO method due to high ISI suppression offered by transmit diversity in the proposed method, and is similar with that after MF combination and adaptive equalization in the SIMO method owing to similar ISI suppression performance of the two methods. The experiment results shown in Figure 11 and Figure 12 are quite consistent with corresponding simulation results.

## 5. Conclusions

In this paper, the joint TR-STBC and adaptive equalization FMT-UWA communication method has been proposed to suppress ISI with simple receive configurations. In the proposed method, the TR-STBC technique designed for frequency-selective fading channels is exploited to replace receive diversity with transmit diversity to offer high diversity gain for good ISI suppression, and adaptive equalization is used as post-processor.

The proposed method is analyzed in theory, and verified by simulation analysis based on shallow channel model and real data collected in the experiment carried out in an indoor pool with multipath propagation. The results show that the proposed method can achieve better communication performance than the joint SISO and adaptive equalization method, and acquire similar performance as the joint SIMO and adaptive equalization method with simpler receive configurations.

## Figures and Tables

**Table 1:** Parameters for simulation analysis.

Parameters	The Proposed Method	SISO Method	SIMO Method
The number of transmit elements	2	1	1
The number of receive elements	1	1	2
Communication band (kHz)	8–16	8–16	8–16
The number of sub-bands of filtered multitone (FMT) modulation	8	8	8
Roll-off factor of each transmit filter	0.5	0.5	0.5
Mapping pattern	Binary phase shift keying (BPSK)	BPSK	BPSK
Number of symbols on each sub-band	1600	1600	1600
Number of training symbols on each sub-band	200	200	200
The number of equalization coefficients on each sub-band	13	13	13
Forgetting factor of recursive least square (RLS)	0.999	0.999	0.999
